# The MOVE study: a study protocol for a randomised controlled trial assessing interventions to maximise attendance at physical activity facilities

**DOI:** 10.1186/s12889-015-1735-0

**Published:** 2015-04-18

**Authors:** Joshua D Newton, Ruth Klein, Adrian Bauman, Fiona J Newton, Ajay Mahal, Kara Gilbert, Leon Piterman, Michael T Ewing, Robert J Donovan, Ben J Smith

**Affiliations:** Department of Marketing, Deakin Business School, Deakin University, 70 Elgar Road, Burwood, VIC 3125 Australia; School of Public Health and Preventive Medicine, Alfred Centre, Monash University, 99 Commercial Road, Melbourne, VIC 3004 Australia; School of Public Health, University of Sydney, Sydney, NSW 2006 Australia; Department of Marketing, Peninsula Campus, Monash University, McMahons Road, Frankston, VIC 3199 Australia; Office of the Pro Vice Chancellor, Peninsula Campus, Monash University, McMahons Road, Frankston, VIC 3199 Australia; Faculty of Health Sciences, Curtin University, Hayman Road, Bentley, WA 6102 Australia

**Keywords:** Physical activity, Physical environment, Intervention trial, Social marketing, Health behaviour

## Abstract

**Background:**

Physical activity is associated with a host of health benefits, yet many individuals do not perform sufficient physical activity to realise these benefits. One approach to rectifying this situation is through modifying the built environment to make it more conducive to physical activity, such as by building walking tracks or recreational physical activity facilities. Often, however, modifications to the built environment are not connected to efforts aimed at encouraging their use. The purpose of the Monitoring and Observing the Value of Exercise (MOVE) study is to evaluate the effectiveness of two interventions designed to encourage the ongoing use of a new, multi-purpose, community-based physical activity facility.

**Methods/design:**

A two-year, randomised controlled trial with yearly survey points (baseline, 12 months follow-up, 24 months follow-up) will be conducted among 1,300 physically inactive adult participants aged 18–70 years. Participants will be randomly assigned to one of three groups: control, intervention 1 (attendance incentives), or intervention 2 (attendance incentives and tailored support following a model based on customer relationship management). Primary outcome measures will include facility usage, physical activity participation, mental and physical wellbeing, community connectedness, social capital, friendship, and social support. Secondary outcome measures will include stages of change for facility usage and social cognitive decision-making variables.

**Discussion:**

This study will assess whether customer relationship management systems, a tool commonly used in commercial marketing settings, can encourage the ongoing use of a physical activity facility. Findings may also indicate the population segments among which the use of such systems are most effective, as well as their cost-effectiveness.

**Trial registration:**

Australian New Zealand Clinical Trials Registry: ACTRN12615000012572 (registered 9 January 2015).

## Background

Physical activity confers a range of health benefits, including reduced risks of developing coronary heart disease, diabetes, and cancers of the breast and colon [1]. Unfortunately, few individuals undertake the levels of physical activity necessary to realise these benefits. Population-level surveys, for instance, indicate that 66.9% of Australians aged 15 years or older are insufficiently active for health [2]. Despite several decades of effort, new and more effective community-based methods for increasing physical activity levels are warranted.

One potential approach for addressing low levels of physical activity is to modify the built environment. Cross-sectional studies have identified a range of environmental features associated with physical activity, including the availability and proximity of recreational facilities and the presence of infrastructure that supports walking, cycling, and public transport use [3,4]. Quasi-experimental research has also found that modifying these environmental features, such as by upgrading park facilities or introducing walking trails and cycling paths, can boost the physical activity of surrounding populations [5].

While there are numerous ways in which the built environment can be modified to encourage greater levels of physical activity, one modification that may deliver particular health benefits for surrounding populations is the development of community physical activity facilities, such as leisure centres, pools, and gyms. For example, economic modelling suggests that gym patronage decreases healthcare spending in Australia by up to $108 million per annum through reductions in the incidence of Type 2 diabetes, cardiovascular disease, and other chronic diseases associated with a lack of physical activity [6]. It has also been estimated that increasing gym patronage by as little as 3% could result in an additional $205 million in annual healthcare savings [6].

Despite the potential health benefits that can accrue from modifying the built environment, many such modifications are implemented with a ‘build it and they will come’ philosophy. That is, once a modification to the built environment has been made, few follow-up interventions aimed at motivating surrounding populations to make use of these modifications are conducted. The extent to which such interventions can further increase the physical activity impacts of modifications to the built environment consequently remains unclear.

This paper describes the protocol of the MOVE (Monitoring and Observing the Value of Exercise) study, a randomised controlled trial designed to assess the effectiveness of two low intensity interventions aimed at maximising attendance at a newly constructed multipurpose physical activity facility. To our knowledge, this will be the first such evaluation to be conducted. It will also be the first longitudinal study to determine whether the introduction of multipurpose recreational facilities influences the physical activity of inactive individuals within the surrounding population.

## Methods/design

### Participants and setting

The MOVE study will be conducted in cooperation with the Peninsula Aquatic Recreation Centre (PARC), a public aquatic and gym facility that opened in September 2014. The facility is located in the City of Frankston, an urban municipality with a population of 126,000 residents that encompasses several outer south-eastern suburbs of Melbourne, Australia [7]. The facility includes a 50 m indoor pool, dedicated learn to swim pools, an aquatic playground area and water slides as well as a spa, sauna, gym, and group exercise rooms.

Approximately 1,300 physically inactive adult participants will be recruited to take part in the study. Physically inactive adults are a priority population group who will experience the greatest benefits from increasing their physical activity levels. Each participant will be assigned to either a control group or one of two intervention groups. Study inclusion and exclusion criteria are outlined in Table 1.

**Table 1:** **MOVE study inclusion and exclusion criteria**

**Inclusion criteria**	**Exclusion criteria**
▪ Reside in Frankston City Council	▪ Unable to walk independently
▪ Aged 18–70 years	▪ Purchased PARC membership
▪ Undertake 30 minutes or more of physical activity sufficient to raise breathing rate on < 5 occasions in a usual week	▪ Poor English skills
▪ Exercise in a recreation or leisure centre, gym, or pool facility on < 3 days in a usual week	

### Funding and ethics approval

Funding for the project has been obtained through the Australian Research Council – Linkage Projects funding scheme (Project ID: LP130101005) and from the linkage partner, Frankston City Council. The project has received ethics approval from the Monash University Human Research Ethics Committee (Project IDs: CF14/1148 – 2014000497 and CF14/2059 – 2014001074) and is registered with the Australian New Zealand Clinical Trials Registry (Trial ID: ACTRN12615000012572).

### Study protocol

**Figure 1:**
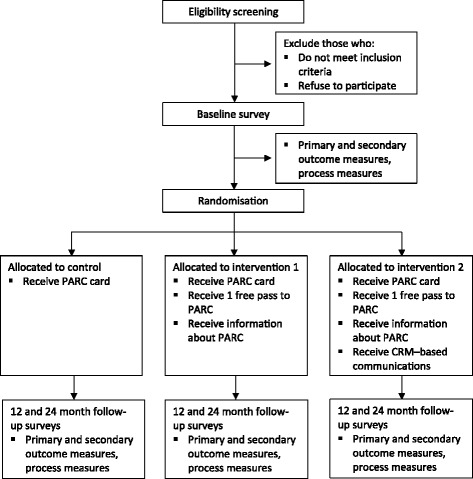
Overview of the MOVE study protocol. Legend: Primary outcome measures include physical activity participation and PARC usage, mental and physical wellbeing, community connectedness and social capital, and friendship and social support. Secondary outcome measures include stages of change for PARC usage, social cognitive decision making variables, health status, and demographic characteristics. Process measures include dose delivered, reach, dose received, and contextual influences upon intervention delivery.

An overview of the proposed study protocol can be found in Figure 1.

#### Recruitment and screening

Participants will be contacted via telephone to determine their eligibility to take part in the study. This telephone contact will be conducted by an independent social research firm and will involve dialling a random selection of landline and mobile phone numbers listed in the Electronic White Pages, with each number belonging to a household within the Frankston City Council area. For each randomly selected telephone number, four to six call attempts at various times of the day will be conducted until contact is made with a member of the household.

Once telephone contact with a household has been made, one household member from among those currently residing within the household will be randomly selected to undertake the screening questions. This will be achieved by asking for household members in the eligible age range to be listed from oldest to youngest, and using computer generated random ordering to identify the first person to invite. If this household member is found to be eligible, they will be briefed about the study aims and invited to take part in the study. If they are not found to be eligible, permission will be asked to screen another member of the household for their eligibility to take part in the study. Eligible individuals who verbally consent to take part in the study will then complete the baseline survey immediately via telephone.

Recruiting participants via telephone directories such as the Electronic White Pages can undersample particular demographic groups that do not have a listed or connected landline telephone number, such as young adults [8]. In an effort to counteract this potential issue, community-based recruitment conducted at shopping centres and community venues located within the Frankston City Council municipality will also be undertaken to maximise the number of young adults taking part in the MOVE study. Individuals passing through these locations will be approached in person and asked to complete the screening questions. As with the telephone recruitment, those found to be eligible will be briefed about the study and invited to become a participant. Those who verbally consent to participate in the study will then be asked to immediately complete the baseline survey.

#### Randomisation and blinding

After the baseline survey has been completed, participants will be assigned to the control group or one of two intervention groups by means of electronic random number generation. Participants will not be blind to the group to which they have been allocated. Similarly, three key researchers (BS, JN, RK) will not be blind to participants’ group allocation as they will be responsible for managing the various study intervention elements and undertaking supplementary qualitative interviews with a subset of participants from each group. However, the research personnel undertaking the follow-up surveys will be blind to the group to which participants will be allocated.

#### Measurement procedure

Individuals who consent to take part in the MOVE study will immediately complete the baseline survey. Specifically, for those contacted via telephone, the baseline survey will be completed using computer-assisted telephone interviewing (CATI). Conversely, those contacted within a community setting will complete the survey face-to-face with a research assistant, as measures completed via face-to-face interviews have been found to have good concurrent agreement with those administered via telephone [9]. All follow-up surveys (i.e., those conducted at 12 and 24 months post-baseline) will be undertaken using the CATI method.

To aid in the recruitment and ongoing retention of participants, incentives will be provided across the course of the study. Participants who complete the baseline survey will automatically receive a chance to win one of three AUD$150 supermarket vouchers. For each post-baseline survey, participants will also receive further chances to win supermarket vouchers. Other episodic incentives in the form of prize draws will also be offered throughout the course of the study to assist in the ongoing retention of participants (e.g., random chance to win a Christmas prize voucher).

#### Intervention procedure

**Figure 2:**
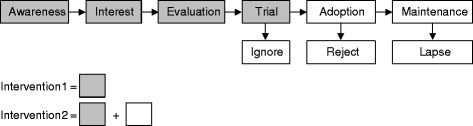
Application of Rogers’ (1962) product adoption process to the study interventions.

The two MOVE study interventions will make use of a social marketing framework [10,11] in that they draw on commercial marketing techniques to promote the adoption of health-related behaviours. Specifically, the interventions are designed to address key phases of Rogers’ [12] adoption process, a model outlining the five typical stages through which individuals move before adopting a new product. These stages are: awareness, interest, evaluation, trial, and adoption (see Figure 2). Awareness, interest, and evaluation are cognitive decision-making processes whereby individuals first become aware of a new product, gain interest in that product, and go on to evaluate the product’s promised benefits. Evaluation may then lead to product trial and (if product trial is successful) product adoption. In physical activity contexts, a sixth stage (maintenance) should also be examined because the health gains associated with physical activity only become evident if this behaviour is sustained over an extended period of time [13].

Participants in both the intervention 1 and intervention 2 groups will receive an information pack describing PARC and the various physical activity facilities available at the centre. This information is designed to target the ‘awareness’, ‘interest’, and ‘evaluation’ phases of Rogers’ [12] product adoption process outlined in Figure 2. Intervention 1 and intervention 2 group participants will also receive a free pass to PARC, allowing them to try out the facility as per the ‘trial’ phase of Rogers’ [12] product adoption process.

**Figure 3:**
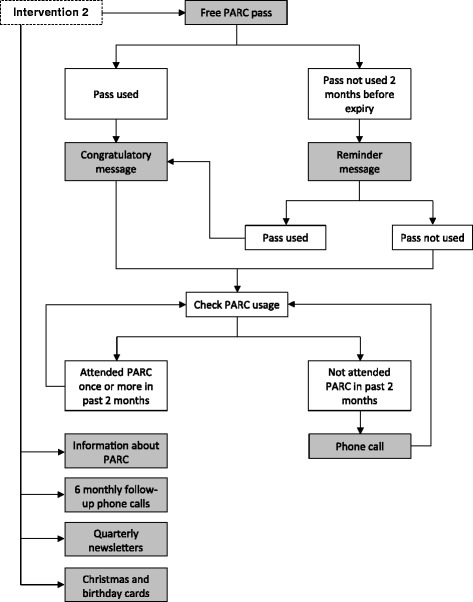
Overview of the customer relationship management (CRM) process for intervention 2 participants. Legend: Grey-coloured boxes denote participant contact points.

Participants in the intervention 2 group will receive additional content designed to target the ‘adoption’ and ‘maintenance’ phases of Rogers’ [12] product adoption process. This content will be delivered over a 24 month period as part of a customer relationship management (CRM) system, a commercial marketing technique designed to manage a firm’s interactions with current and future customers [14] and which has been found to maximise customer loyalty and retention [15]. An overview of the key CRM contact points with intervention 2 participants is presented in Figure 3. Specifically, upon redeeming the free PARC pass, intervention 2 participants will receive a message congratulating them for redeeming their pass and encouraging them to return to the centre soon. Two months before the pass expires, those who are yet to redeem the free pass will receive a telephone call reminding them that the pass will be expiring soon. Finally, those who have attended PARC at least once but who have not returned to the facility in the last two months will receive a follow-up telephone call to encourage their continued use of the facility.

Several other contact points will be integrated into the CRM system. For example, intervention 2 participants will receive follow-up phone calls every 6 months to discuss their physical activity goals, identify strategies for overcoming barriers to physical activity, and encourage their usage of PARC. Intervention 2 participants will also receive a quarterly newsletter aimed at motivating regular physical activity and identifying strategies to overcome common barriers to engaging in regular physical activity. Embedded within the newsletter will be content espousing the benefits of conducting regular physical activity at PARC. Finally, participants will receive personalised, hand-written cards for their birthday and in the lead-up to the end of year festive season. Each card will encourage recipients to achieve their physical activity goals.

### Outcome measures

#### Primary outcome measures

Primary outcome measures will be PARC usage, physical activity participation, mental and physical wellbeing, community connectedness, social capital, friendship, and social support.

##### Physical activity participation and PARC usage

The Exercise Recreation and Sport survey [16] will be used to measure participation in organised and non-organised leisure activities during the past 12 months and 2 weeks, respectively [17]. This measure will also be used to derive a summary measure of physical activity participation [18]. Facility-based physical activity will be also tracked by means of a study-specific swipe card, which all participants will be encouraged to use each time they pay for entry to PARC. If any participant goes on to purchase a long-term membership to PARC during the course of the study, their new PARC membership card will be linked (via the PARC customer database) to their original study-specific swipe card. These individuals will then only need to display their PARC membership card upon entry to the facility for their attendance to be recorded. Finally, self-reported PARC usage will be assessed in the two post-baseline follow-up surveys.

##### Mental and physical wellbeing

Mental and physical wellbeing will be assessed using the Warwick Edinburgh Mental Well-Being scale [19] and two single-item measures [20,21]. The Warwick Edinburgh Mental Well-Being scale is a 14-item measure that provides a single score assessing fundamental elements of mental health, such as happiness, self-realisation, positive affect, satisfying interpersonal relationships, and functioning. Psychometric testing indicates that the scale has high internal consistency, acceptable test-retest reliability, and good concurrent validity with a range of other mental health and well-being scales [19]. Two single-item measures will also be used to assess global mental and physical health. The first, global self-rated mental health [20], captures emotional wellbeing and role functioning, while the second, global quality of life [21], has concordance with the EQ-5D measure of health outcomes [22].

##### Community connectedness and social capital

Two items taken from the Australian Unity Wellbeing Index [23] will be used to measure community relationships (i.e., community connectedness) and sense of trust in others (i.e., social capital). Each item has been reported to have construct validity with respect to other measures of life satisfaction and community wellbeing [23].

##### Friendship and social support

Friendship and social support will be assessed using the six-item Friendship Scale [24]. This scale is reported to have good internal reliability and concurrent validity when assessed against other short social relationship scales [24].

#### Secondary outcomes measures

A range of secondary outcome measures will also be assessed and examined as potential meditators and moderators of the primary outcomes.

##### Stages of change for PARC usage

A single-item measure of stage of readiness to attend PARC will be developed by modifying an existing measure of stage-of-change for physical activity that has been reported to have good construct validity and test-retest reliability [25]. The original item will be modified by replacing references to “exercise” with the term “attend PARC”.

##### Social cognitive decision making

A theory of planned behaviour framework [26,27] will be used to examine participants’ social cognitive decision-making around performing regular physical activity. Specifically, single-item scales adapted from a range of sources will be used to assess the following constructs: intention [28], attitude [29], subjective norm [29], and self-efficacy [30]. Anticipated regret [31] and action planning [32], two constructs that are compatible with a theory of planned behaviour framework, will also be assessed. All items will adhere to the measurement guidelines advanced by Fishbein and Ajzen [27], particularly with respect to ensuring that the action, target, context, and time of the behavioural criterion (i.e., regular physical activity) are compatible across all items.

##### Health status and demographic characteristics

A short version of the Functional Comorbidity Index, which has been validated in a variety of contexts [33], will be used to measure current comorbidities (e.g., arthritis, diabetes). Self-reported residential address will be used to determine proximity by road to PARC. Other demographic variables will also be collected, including sex, age group, household structure, educational attainment, occupation, household income, Aboriginality, country of birth, and language spoken at home.

#### Process evaluation measures

The process measures will follow the recommendations of Steckler and Linnan [34] and examine dose delivered, reach, dose received, and contextual influences upon intervention delivery. The CRM database, which will be constructed using Filemaker Pro, will document when each component of the intervention program is delivered to intervention participants. Participant demographic information collected at baseline will be used to ascertain the representativeness of each group with respect to the wider Frankston City Council population. Intervention 1 and 2 participants’ usage of the free PARC passes will be recorded. Dose received will also be measured by asking Intervention 2 participants at each follow-up survey point about their use and recall of the CRM materials. In addition, qualitative semi-structured interviews will be conducted with 15 control, 15 intervention 1, and 15 intervention 2 participants between 10 and 18 months of the opening of PARC. These interviews will seek to explore barriers and facilitators to their use of PARC. Participants in the intervention 2 group will also be asked their thoughts about the CRM messages they received during the course of the study. Participants will be purposively sampled from the larger study cohort to ensure both regular users and non-users of PARC from each group are represented.

### Statistical considerations

#### Sample size

Sample size calculations have been undertaken to determine the number of participants needed to show a significant difference in attendance rates at PARC. To be conservative in sample size calculations (i.e., avoid lack of power), it is assumed that 10% of control group participants will become regular users of PARC. Thus, a sample size of 300 in each arm of the study will be required to show with 95% confidence limits and 80% power: (i) a 10% difference in outcome between participants in intervention 1 and the control group (i.e., 20% vs. 10%); and (ii) a 10% difference between those in intervention 1 and intervention 2 (i.e., 30% vs. 20%). If fewer than 10% of control group participants attend PARC regularly, this sample size will enable detection of smaller differences between participants in the control and intervention groups. To accommodate attrition over the course of the two year study, the target sample size will be inflated to 400 per group for the two intervention groups and 500 for the control group because of the potential reduced engagement that control group participants may have with the study.

#### Data analysis

Process data about intervention dose delivered, reach, and dose received will be summarised using descriptive statistics. Transcripts from semi-structured interviews about the contextual factors affecting use of PARC and satisfaction with the CRM activities will be analysed using thematic analysis [35]. Bivariate and multivariate statistical tests will be used to analyse differences between the control, intervention 1, and intervention 2 groups on the primary outcome measures across the three data collection points. In the multivariate analyses, group status (i.e., control, intervention 1, intervention 2) will be entered as a covariate to calculate intervention effect sizes. Mediation and moderation analyses [36] will also be used to examine whether the secondary outcome variables influence the intervention effect sizes. Analysis will be undertaken by intention to treat and by treatment received according to the dose received of marketing components.

#### Economic evaluation

Cost-effectiveness analyses for interventions 1 and 2 will compare gains in the primary outcome variables relative to controls, taking into account the additional costs associated with implementing these interventions. In addition, the impact of interventions 1 and 2 on PARC membership and membership renewal, once participation incentives end, will be used to explore the potential implications of marketing interventions for promoting and maintaining memberships and revenues.

## Discussion

A large proportion of the Australian population do not meet the recommended levels of physical activity, thereby forgoing the protective health benefits that physical activity confers. Strategies aimed at increasing physical activity levels, such as encouraging surrounding populations to make use of new physical activity facilities or other changes to the built environment, are therefore of particular importance. The MOVE study will consequently assess whether two commercial marketing approaches are equally effective in motivating communities to engage with and utilise a new pool and gym facility. This is to our knowledge the first such evaluation to be undertaken and represents a central contribution of the MOVE study.

Leveraging commercial marketing techniques to improve the wellbeing of individuals or communities (i.e., ‘social marketing’ [37]) is not unprecedented, with such techniques playing an important role in addressing a range of public health challenges [38]. What has yet to be examined is whether these techniques can be effectively utilised to encourage inactive populations to make use of new physical activity facilities. This is particularly true for CRM, a commercial marketing technique that has been recognised as having potential efficacy in social marketing contexts [39], but which has yet to have these novel applications empirically tested. Comparing the efficacy of CRM and non-CRM social marketing interventions within the context of encouraging ongoing attendance at physical activity facilities therefore represents a key innovation of the MOVE study.

A second innovative feature of the MOVE study relates to segmentation, another principle widely employed in social marketing settings [11]. Segmentation represents an acknowledgement that certain groups within a population may share similarities on various social, psychological, or demographic dimensions, and that developing interventions that target one or more of these groups may increase the efficacy of those interventions [11,40]. The MOVE study will utilise a post-intervention segmentation approach in that it will test for potential moderators of intervention effectiveness to determine whether some population groups may be particularly responsive to the interventions being tested. This, in turn, could guide future decisions about which population groups to preferentially target in interventions.

A third innovative feature of the MOVE study is the emphasis on developing financially sustainable interventions. The initiation and continuation of public health programs is often reliant upon government or philanthropic funding, potentially jeopardising the longer-term viability and impact of these programs if governments change or funding priorities shift. Furthermore, in an environment of escalating healthcare costs [41], it is important that financially sustainable methods for achieving community-wide changes in health are identified. To this end, the proposed interventions are ultimately designed to be adopted by another, oft-overlooked source of public health funding: the operators of physical activity facilities such as pools and gyms. While these operators share an interest in encouraging the adoption of healthier lifestyles, they often lack the tools or knowledge necessary to successfully engender behaviour change at a community-wide level. Moreover, the operators of physical activity facilities have traditionally focused on acquiring new members as opposed to supporting and retaining existing members [42]. The proposed project will therefore assess the efficacy of two approaches that could feasibly be integrated into the marketing plans of physical activity facilities around Australia. This, in turn, maximises the likelihood that the intervention elements will be sustained beyond the life of the project and adopted in other localities.
